# Assessment of the Length of the Pedicle of the Anterolateral Thigh Flap

**DOI:** 10.7759/cureus.54836

**Published:** 2024-02-24

**Authors:** Bartłomiej Wilk, Ewa Tramś, Marcin Zlotorowicz, Kamil Kołodziejczyk, Ewa Nosarzewska, Jarosław Czubak

**Affiliations:** 1 Orthopaedics and Traumatology, Medical University of Warsaw, Warsaw, POL; 2 Orthopaedics, Pediatric Orthopaedics and Traumatology, Gruca Teaching Hospital, Otwock, POL

**Keywords:** lfca, lateral femoral circumflex artery, pedicle, alt, anterolateral thigh flap

## Abstract

Purpose

To estimate the length of the pedicle of the anterolateral thigh flap (ALT) and to assess how this length corresponds with the distances between the anatomical landmarks.

Methods

The study group consisted of patients who underwent computed tomography angiograms ranging minimally from the anterior superior iliac spine (ASIS) superiorly and tibia inferiorly. In the study we included 44 patients. In the axial window we identified single scans with (1) ASIS, (2) the apex of the greater trochanter, (3) the origin of the descending branch of the lateral femoral circumflex artery (LFCA), (4) the superolateral corner of the patella, (5) knee joint gap. Knowing the slice thickness in every patient and the difference in scan number we measured (A)-the distance between the scan (1) and the scan (4). This distance (A) represented the length of the line connecting ASIS and the superolateral corner of the patella (AP line). Next, we identified (6) the midpoint of the distance (A). Next we measured (B)-the distance between the scan (2) and the scan (5) and (C)-the distance between the scan (3) and the midpoint of the AP line (6).

Results

Mean distances between the scans were: (A) 45.34 cm (SD=4.14), (B) 43.12 cm (SD=4.08), (C) 11.69 cm (SD=1.62). There was low positive correlation between the distance (A) and the distance (C) (r_s_=0.43) and moderate positive correlation between the distance (B) and the distance (C) (r_s_=0.53).

Conclusion

Our study suggests that the mean estimated length of the ALT flap pedicle is 11.69 cm and that it positively correlates with the length of the femur and the length of the AP line.

## Introduction

Anterolateral thigh flap (ALT) was first described by Song et al. in 1984. The authors of the study reported the possibility of using a septocutaneous flap with blood supply from the descending branch of the lateral femoral circumflex artery (LFCA) [1]. Following studies have proven that in most patients septocutaneous perforator for the ALT flap cannot be found resulting in the need to use a musculocutaneous perforator which requires a more challenging dissection technique [2-6]. Those reports combined with the fact that there are several anatomical variations of the LFCA [2,7] may be the reason why this particular flap is not chosen by some surgeons.

On the other hand, the ALT flap has many advantages. The flap has a relatively wide pedicle thus anastomosis can be easily performed [1,8]. What is more, the flap can cover a large surface. Studies report a maximal length of the flap of 35 cm and a maximal width of 21 cm [2]. There are some reports of complications following the operation such as range of motion (ROM) limitation, sensory disturbance and fatigue in the donor-site limb [9]. However, studies prove that the overall success rate is higher than 90% [6,10] and the satisfaction rate is as high as 87.5% [9]. Demirkan et al. call the ALT flap “versatile and dependable” and a review conducted by Graboyes et al. describing novel uses of the flap seems to support this statement [10,11]. The regional alternative flap options for patients lacking suitable perforators for the ALT flap are the tensor fasciae latae flap and the anteromedial thigh flap [12].

A commonly described advantage of the ALT flap is its long vascular pedicle [5,6,11], although the data about exact pedicle length is not consistent. Originally Song et al. described the pedicle to be longer than 8 cm [1]. Begue et al. in their anatomical studies state that the specific pedicle is 5 cm long but it could be increased to 10 cm by ligature of the branches for the vastus lateralis muscle [13]. Pribaz et al. suggest that the pedicle of the flap can be up to 15 cm long when dissecting the origin of the LFCA [6]. Kimata et al. in their report of 74 cases write that the pedicle length depends on the selection of the perforator [2]. There are three described concentration points of perforators that could be used for the ALT flap. A useful tool in the identification of the concentration points is the line connecting the anterior superior iliac spine (ASIS) and the superolateral corner of the patella (AP line). The perforators concentrate near the AP line in the (1) Point in the proximal 0.4 of the AP line, (2) Midpoint of the AP line, (3) Point in the distal 0.6 of the AP line [4]. Most of the studies agree that among these points the midpoint of the AP line is the point of the highest concentration of the ALT flap perforators [2,4,5,10].

There is a significant need for a study that would help to assess the length of the pedicle of the ALT flap since previously collected data varies when it comes to measurement methods and acquired results.

The aim of the study was to create a method that would allow estimation of the length of the pedicle of the ALT flap and examine how the measurements made with this method correspond with the distances between the anatomical landmarks.

## Materials and methods

We designed and conducted an analytical, observational study. The data of consecutive patients who underwent computed tomography angiograms were retrospectively analyzed. All of the computed tomography angiograms were obtained with Toshiba Aquilion One TSX-301A, 320 Slice Acquisition (Toshiba Medical Systems, Tokyo, Japan). The standard protocol was utilized for pelvis and lover extremity computed tomography angiograms (120 kV, auto-mA maximal 300 mA). The details of the protocol varied depending on the examined patient and on the cause of the examination. The study group consisted of patients who underwent computed tomography angiograms ranging minimally from ASIS superiorly and tibia inferiorly. We included only the angiograms with a slice thickness of less than 1 mm for maximal precision when it comes to identifying the exact location of arterial division. The studies with artifacts that would compromise the assessment of the arteries were excluded. We excluded angiograms with sclerotic changes visible.

After considering these criteria we included in the study 44 patients. The group consisted of 30 men and 14 women with a mean age of 65.9 years (range: 47-87). In every patient we individually analyzed both the left and the right side (a total of 88 analyzed lower extremities - 44 left and 44 right).

In the axial window we identified single scans with (1) ASIS, (2) the apex of the greater trochanter, (3) the origin of the descending branch of LFCA, (4) the superolateral corner of the patella, and (5) the knee joint gap.

**Figure 1:**
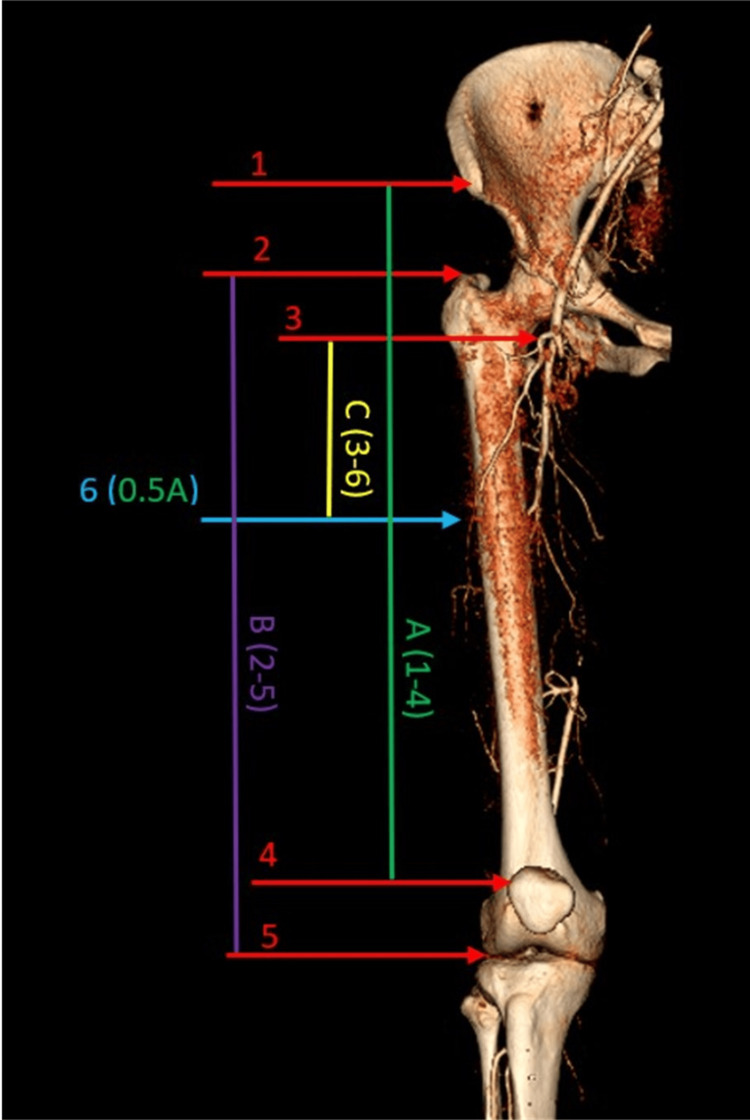
Measurements. Representation of the measurements taken in the study on a 3D computed tomography reconstruction. (1) the scan with ASIS, (2) the scan with the apex of the greater trochanter, (3) the scan with the origin of the descending branch of LFCA, (4) the scan with the superolateral corner of the patella, (5) the scan with the knee joint gap, (A) the distance between the scans (1) and (4), (6) the midpoint of the distance (A), (B) the distance between the scans (2) and (5), (C) the distance between the scan (3) and the midpoint (6). The anterior layers of the reconstruction are removed for better visualization of LFCA. ASIS: anterior superior iliac spine; LFCA: lateral femoral circumflex artery

Knowing the slice thickness, which was 0.5 mm in every patient, and the difference in scan number we measured the following: (A) the distance between (1) the scan with ASIS and (4) the scan with the superolateral corner of the patella. This distance (A) represented the length of the AP line. Next, we identified (6) the midpoint of (A) the distance between (1) the scan with ASIS and (4) the scan with the superolateral corner of the patella representing the midpoint of the AP line; (B) The distance between (2) the scan with the apex of the greater trochanter and (5) the scan with the knee joint gap. This distance (B) represents the length of the femur; (C) The distance between (3) the scan with the origin of the descending branch of LFCA and (6) the midpoint of the distance (A) between (1) the scan with ASIS and (4) the scan with the superolateral corner of the patella. We assume that this distance (C) could serve as the approximate length of the ALT flap pedicle since it is the distance between the origin of the branch of the artery that gives blood supply for the ALT flap and the point of the highest concentration of the ALT flap perforators. The measurements are depicted in Figure 1.

Statistical analysis of acquired data was performed using TIBCO Software Inc. Statistica Version 13.3 for Windows. Shapiro-Wilk test was used to examine the normal distribution of parameters. Spearman’s rank order correlation coefficient was used to measure the association between given variables. The significance level was set at a p value of 0.05.

The procedures performed were in accordance with the ethical standards of the Institutional Scientific Committee (IRB) as well as with the 1964 Helsinki Declaration and its later amendments. No IRB approval was required for this study.

## Results

**Figure 2:**
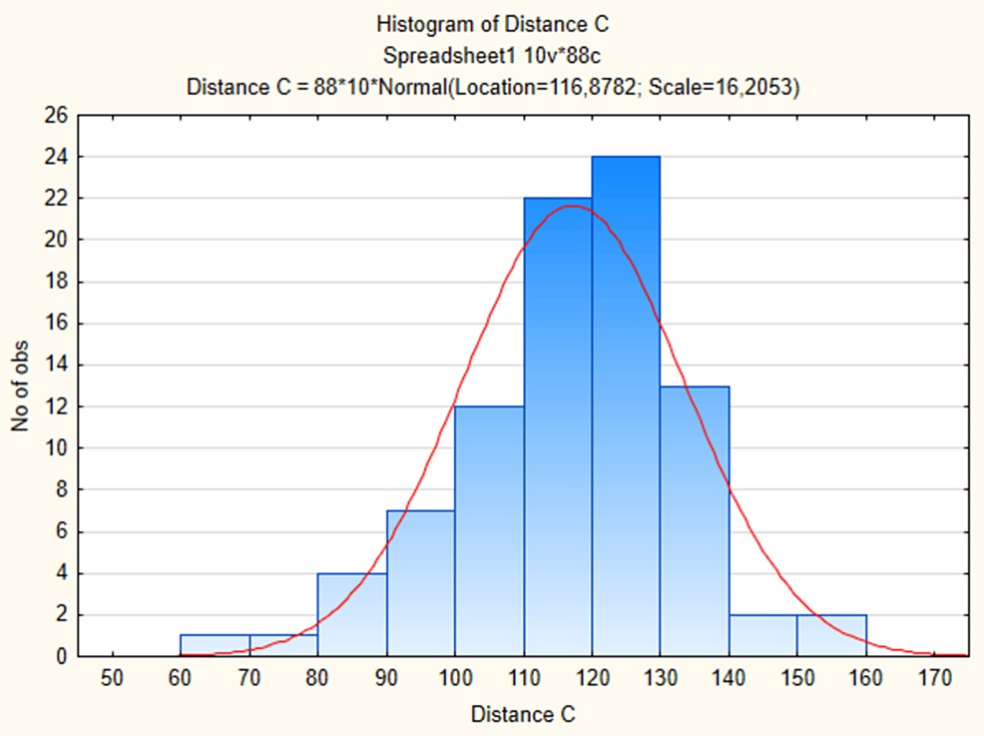
Distribution of the distance (C). The histogram representing the distribution of the distance (C) between (3) the scan with the origin of the descending branch of LFCA and (6) the midpoint of the distance (A) between (1) the scan with ASIS and (4) the scan with the superolateral corner of the patella (in millimeters). The histogram compares the data to normal parameter distribution (the red line). X-axis: the length of the pedicle of the ALT flap (Distance (C); in millimeters), Y-axis: number of observations. ASIS: anterior superior iliac spine; LFCA: lateral femoral circumflex artery

Mean distance (A) between (1) the scan with ASIS and (4) the scan with the superolateral corner of the patella was 45.34 cm (SD=4.14) (Table 1). Mean distance (B) between (2) the scan with the apex of the greater trochanter and (5) the scan with the knee joint gap was 43.12 cm (SD=4.08) (Table 1). Mean distance (C) between (3) the scan with the origin of the descending branch of LFCA and (6) the midpoint of the distance (A) between (1) the scan with ASIS and (4) the scan with the superolateral corner of the patella was 11.69 cm (SD=1.62) (Table 1, Figure 2).

**Table 1:** Results. The table depicts the results of the study: Mean (in centimeters), Standard deviation (Std. Dev.) and Spearman’s Rank Order Correlation (all bolded results are significant at p<0.05). (A) the distance between (1) the scan with ASIS and (4) the scan with the superolateral corner of the patella, (B) the distance between (2) the scan with the apex of the greater trochanter and (5) the scan with the knee joint gap, (C) the distance between (3) the scan with the origin of the descending branch of LFCA and (6) the midpoint of the distance (A). ASIS: anterior superior iliac spine; LFCA: lateral femoral circumflex artery

Results			Spearman Rank Order Correlations (p<0.05)		
Variable	Mean (cm)	Std. Dev.	Distance (A)	Distance (B)	Distance (C)
Distance (A)	45.34	4.14	-------------------	0.87	0.43
Distance (B)	43.12	4.08	0.87	-------------------	0.53
Distance (C)	11.69	1.62	0.43	0.53	-------------------

Shapiro-Wilk test has shown that only the distance (A) has normal parameters distribution. Thus Spearman Rank Order Correlation was used to assess the relation between the variables.

There was a low positive correlation between the distance (A) between (1) the scan with ASIS and (4) the scan with the superolateral corner of the patella and the distance (C) between (3) the origin of the descending branch of LFCA and (6) the midpoint of the distance between (1) the scan with ASIS and (4) the scan with the superolateral corner of the patella (rs=0.43) (Table 1).

**Figure 3:**
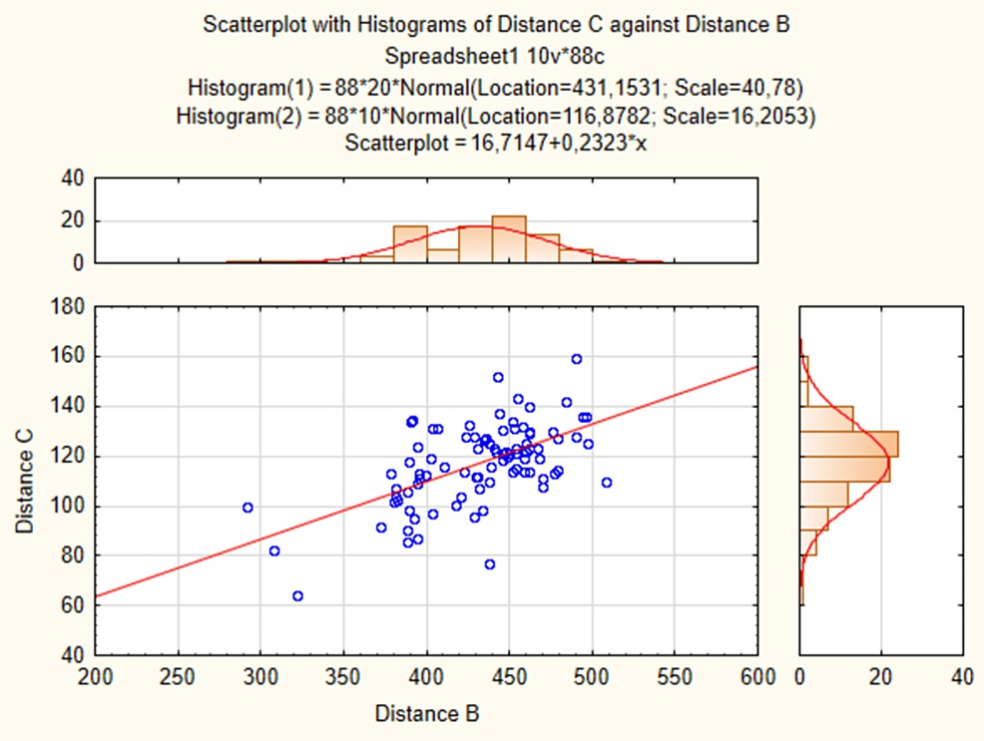
The relation between the distance (B) and the distance (C). The scatterplot with histograms representing the relation between the distance (B) between (2) the scan with the apex of the greater trochanter and (5) the scan with the knee joint gap, and the distance (C) between (3) the scan with the origin of the descending branch of LFCA and (6) the midpoint of the distance (A) between (1) the scan with ASIS and (4) the scan with the superolateral corner of the patella (in millimeters). The scatterplot compares the data to normal parameters distribution (red line). ASIS: anterior superior iliac spine; LFCA: lateral femoral circumflex artery

There was a moderate positive correlation between the distance (B) between (2) the scan with the apex of the greater trochanter and (5) the scan with the knee joint gap and the distance (C) between (3) the origin of the descending branch of LFCA and (6) the midpoint of the distance between (1) the scan with ASIS and (4) the scan with the superolateral corner of the patella (rs=0.53) (Table 1, Figure 3). There were no statistically significant differences in the length of the pedicle of the ALT flap between the right and left sides.

## Discussion

The ALT flap gained popularity since it was first described in 1984 [1]. Unfortunately, contrary to its advantages such as wide and long pedicle, large surface, and high post-operative satisfaction rate the ALT flap may be challenging to use due to anatomical variations of the pedicle. Nevertheless, the flap is commonly used and authors still try to investigate its anatomy, applications and drawbacks to optimize the operative technique.

Our study seems to prove that the length of the AP line correlates to a low degree with the estimated length of the pedicle of the ALT flap and that the length of the femur moderately correlates with the estimated length of the pedicle of the ALT flap. The next conclusion is that the approximate average length of the pedicle for the ALT flap measured with our method is 11.69 cm.

The anatomy of the ALT flap is also an aspect that is still being studied. A recent cadaver dissection study conducted by Gholami et al. explored the variability of ALT flap anatomy. The authors had similar parameters in their scope to the ones we had in our study. They measured the length of the AP line and the length of the vascular pedicle from the cut point to the first perforator. The average length of the AP line measured in this study was 44.6 cm. In our study, the value of this parameter was 45.34 cm. This difference seems to be not relevant and may be caused either by ethnic differences in examined groups or the difference in the size of the study. The cadaver dissection study allowed the authors to examine only 10 lower extremities from 10 cadavers whereas we were able to compare 88 lower extremities from 44 patients. The length of the vascular pedicle from the cut point to the first perforator measured by the authors was 10.17 cm. We did not measure the same parameter however we determined the distance between the origin of the descending branch of LFCA and the midpoint of the AP line achieving the mean value of 11.69 cm. The difference in this measurement may be due to the fact that in the cadaver study the authors assessed the length of the pedicle to the first perforator while for our study the distal point for estimating the length of the pedicle was the midpoint of the AP line. Another reason may be that the authors measured the real length of the pedicle while our study is based on the estimation of this length [14]. However other studies seem to be more consistent with our outcomes reporting ALT flap pedicle to be 11.4 cm long [15].

Authors investigate various preoperative planning methods to assess the ALT flap pedicle. Łuczewski et al. propose a mathematical formula based on preoperative Doppler sonography to predict the length of the pedicle of the ALT flap. The authors claim that the intraoperatively measured pedicle length was equal or larger than the estimated length in 73.9% of the cases [16]. However, the estimated length agreed with intraoperative findings only in 14.54% of the cases. In our study, we did not have the opportunity to compare the estimated pedicle length with the intraoperative findings. We consider performing similar research in the future to compare the estimations with the intraoperatively measured pedicle length. Hu et al. examined utilizing magnetic resonance angiography in the ALT flap preoperative planning. The authors compared magnetic resonance angiography imaging findings with intraoperative findings in 19 patients. The method allowed prediction of the course and type of perforators in 94.7% of the cases. Unfortunately, the authors did not examine this method in predicting the ALT flap pedicle length [17]. Nevertheless, magnetic resonance angiography should be considered for further research in this field since the method does not come with patients’ radiation exposure. It is still to be determined whether computed tomography planning should be a standard preoperative technique. The method offers potential benefits such as the ability to track the three-dimensional route of the pedicle, although it also comes with radiation exposure to the patients.

Presented studies depict that the ALT flap is still widely utilized. Authors try to search for new ways to optimize the outcomes by exploring the anatomy of the flap. The research in this field brings new opportunities for safer, more efficient and more versatile surgical techniques adopting the ALT flap. We hope that our study will be the next step in achieving this goal.

In our study, we can identify some advantages in comparison to other studies focused on the ALT flap. Firstly, the method allowed us to compare a relatively large group. On computed tomography angiograms we analyzed 88 lower extremities in 44 patients. In other studies, where the authors chose to dissect the ALT flap from cadavers, the size of the examined group was remarkably smaller [13,14]. Secondly, the method applied in our study allows for precise and consistent identification of important anatomical landmarks. In computed tomography angiograms we were able to track blood vessels and identify them in relation to surrounding structures. Finally, our study is based on an easy and well-described method. This is important because other authors will be able to recreate our measurements for research purposes or to include them in the preoperative planning.

When it comes to limitations, in the study we assume that the midpoint of the AP line is the point where the perforator is most likely to appear. This is well proven in other studies [2,10,11,18]. However, to achieve maximal precision in measuring perforator length, knowing that perforators have anatomical variations, we should map the exact point where the perforator appears in every individual patient. Perhaps a study utilizing computed tomography and Doppler ultrasound would be more accurate. Next, when measuring the length of the perforator we did not factor in the three-dimensional route of the perforator. From our study, we can only assess the length of the perforator from the length of a straight line from the origin of the perforator to the midpoint of the AP line. To obtain the exact length of the perforator we would have to track the perforator location on every scan and then measure its length considering the three-dimensional route. However, this would be extremely hard or even impossible because of the small diameter of the perforator. Blood vessels of such caliber as the distal part of the perforator are poorly visible on computed tomography angiograms.

## Conclusions

The length of the AP line positively correlates in a low degree with the estimated length of the pedicle of the ALT flap. The length of the femur moderately positively correlates with the estimated length of the pedicle of the ALT flap. The approximate average length of the pedicle for the ALT flap calculated with our method is 11.69 cm.
